# Prevalence of central obesity and its associated risk factors among adults in Southeast Ethiopia: A community-based cross-sectional study

**DOI:** 10.1371/journal.pone.0265107

**Published:** 2022-08-05

**Authors:** Yohannes Tekalegn, Damtew Solomon, Biniyam Sahiledengle, Tesfaye Assefa, Wogene Negash, Anwar Tahir, Tadele Regassa, Ayele Mamo, Habtamu Gezahegn, Kebebe Bekele, Demisu Zenbaba, Alelign Tasew, Fikreab Desta, Daniel Atlaw, Zegeye Regassa, Fikadu Nugusu, Zinash Teferu Engida, Degefa Gomora Tesfaye, Chala Kene, Wondu Shiferaw Nigussie, Dereje Chala, Adisu Gemechu Abdi, Girma Beressa, Demelash Woldeyohannes, Heather L. Rogers, Lillian Mwanri

**Affiliations:** 1 Public Health Department, Goba Referral Hospital, Madda Walabu University, Bale Goba, Ethiopia; 2 Biomedical Department, Goba Referral Hospital, Madda Walabu University, Bale Goba, Ethiopia; 3 Nursing Department, Goba Referral Hospital, Madda Walabu University, Bale Goba, Ethiopia; 4 Pharmacy Department, Goba Referral Hospital, Madda Walabu University, Bale Goba, Ethiopia; 5 Surgery Department, Goba Referral Hospital, Madda Walabu University, Bale Goba, Ethiopia; 6 Midwifery Department, Goba Referral Hospital, Madda Walabu University, Bale Goba, Ethiopia; 7 School of Medicine and Health Science, Department of Public health, Wachemo University, Hosana, Ethiopia; 8 Biocruces Bizkaia Health Research Institute, Barakaldo, Bizkaia, Spain; 9 Ikerbasque Basque Foundation for Science Bilbao, Bilbao, Bizkaia, Spain; 10 Torrens University Australia, Adelaide, South Australia, Australia; University of Zurich, SWITZERLAND

## Abstract

**Background:**

Obesity and overweight are known public health problems that affect populations across the world. These conditions have been associated with a wide range of chronic diseases including type 2 diabetes mellitus, cardiovascular disease, and cancers. In Ethiopia, the literature regarding the burden of central (abdominal) obesity is scarce. This study aimed to fill this gap by assessing the prevalence and risk factors associated with central obesity among adults in Ethiopia.

**Methods:**

From May to July 2021, a community-based cross-sectional survey was conducted on a sample of 694 adults aged ≥18 years in administrative towns of Bale zone, Southeast Ethiopia. Multi-stage sampling followed by systematic random sampling was employed to identify study participants. Waist and hip circumferences were measured using standard protocols. The World Health Organization STEPS wise tool was used to assess risk factors associated with central obesity. Bi-variable and multi-variable binary logistic regression were used to identify factors associated with central obesity. Adjusted odds ratios (AOR) and their corresponding 95% confidence intervals (CI) have been reported to estimate the strength of associations.

**Results:**

The overall prevalence of central obesity using waist circumference was 39.01% [(95% CI: 35.36–42.76; 15.44% for men and 53.12% for women)]. Multi-variable binary logistic regression analysis revealed that female sex (AOR = 12.93, 95% CI: 6.74–24.79), Age groups: 30–39 years old (AOR = 2.8, 95% CI: 1.59–4.94), 40–49 years (AOR = 7.66, 95% CI: 3.87–15.15), 50–59 years (AOR = 4.65, 95% CI: 2.19–9.89), ≥60 years (AOR = 12.67, 95% CI: 5.46–29.39), occupational status like: housewives (AOR = 5.21, 95% CI: 1.85–14.62), self-employed workers (AOR = 4.63, 95% CI: 1.62–13.24), government/private/non-government employees (AOR = 4.68, 95% CI: 1.47–14.88), and skipping breakfast (AOR = 0.46, 95% CI: 0.23–0.9) were significantly associated with central obesity.

**Conclusions:**

Abdominal obesity has become an epidemic in Bale Zone’s towns in Southeastern Ethiopia. Female sex, age, being employed were positively associated with central obesity, while skipping breakfast was a protective factor.

## Introduction

The World Health Organization (WHO) defines obesity as an abnormal or excess fat accumulation that may impair health [1]. Obesity has been associated with the imbalance between intake and energy expenditure, a condition that may be caused by either non-modifiable or modifiable risk factors [2]. This caloric imbalance creates an excess accumulation of energy, which in turn is stored in the body resulting in excess body weight. Although genetic factors are not modifiable, obesity can result from a complex interaction between environmental, socio-economic, and/or personal behaviors. Addressing the modifiable factors play as one of the critical strategies in preventing obesity [3]. Globally, the prevalence of overweight and obesity increased by 27.5% for adults and 47.1% for children between 1980 and 2013. The number of individuals with overweight and obesity increased from 857 million in 1980 to 2.1 billion in 2013 [4]. If this secular trend continues persistently, 38% of the world’s adult population will be overweight and another 20% will be obese by 2030 [5].

Worldwide, a high body mass index (BMI) has been reported as responsible for four million deaths and 120 million disability-adjusted life years [6]. Several studies have further documented obesity as the risk factor for many non-communicable diseases (NCDs) and chronic health conditions including hypertension, high lipid concentrations, type 2 diabetes, coronary heart disease, stroke, and certain cancers [7–22]. Central (abdominal) obesity, measured in waist circumference, waist to hip ratio, and waist to height ratios, is highly linked with increased risk of morbidity and mortality and is considered to be superior to BMI in predicting cardiovascular disease and mortality risks [23–25].

Many low and middle-income countries (LMICs), including Ethiopia, currently face a double burden of malnutrition [26]. While LMICs are dealing with problems of infectious diseases and undernutrition, they also experience a rapid increase in non-communicable disease risk factors including overweight and obesity [1, 27–29]. The emergence of the Coronavirus Disease (COVID-19) pandemic has further challenged health systems, economies, and populations across the globe, with LMICs being severely affected [30, 31]. Evidence suggests that NCDs are dramatically increasing in Ethiopia and it was estimated that NCDs were responsible for 711 deaths per 100,000 population in 2015 [32]. Cardiovascular diseases, cancer, diabetes, and mental disorders were reported to be responsible for 30% of the total disease burden in Ethiopia as measured in age-standardized disability-adjusted life years (DALYs) rates in 2017 [33].

In Ethiopia, epidemiological studies regarding the prevalence, distribution, and determinants of obesity are meager. More specifically, few studies have assessed the prevalence and risk factors of central (abdominal) obesity [34–39], a superior predictor of NCDs. The prevalence reported in the aforementioned previous studies ranges from 15.5% in the northern part of Ethiopia [36] to 37.4% in southwest Ethiopia [39]. Similarly, risk factors identified by those studies vary from district to district. As a result of this diversity, it is necessary to study the prevalence and context-based risk factors in different settings which will have local and national implications for prevention efforts / public health campaigns to address obesity and other chronic diseases. To the best of our knowledge, there is no published evidence on the magnitude and risk factors associated with central obesity in Southeast Ethiopia. The current study aims to assess the prevalence of central (abdominal) obesity and its associated risk factors in the administrative towns of Bale Zone, Southeast Ethiopia.

## Methods and materials

### Study design, setting, and subjects

From May to July 2021, a community-based cross-sectional study was conducted to assess the prevalence of central (abdominal) obesity among adults (≥18 years) in the administrative towns of Bale zone, Southeast Ethiopia. All adults residing in the study area for at least 6 months were eligible for inclusion. However, potential participants were excluded if they had: psychiatric problems, hearing impairments, body deformities (kyphosis and scoliosis), other debilitations, and/ or handicaps. Pregnant women were also ineligible for inclusion. Bale zone, one of the Zonal administrative units in the Southeastern part of Oromia regional state (an area of 43,690.56 km^2^), is located between 5^0^ 22’– 8^0^ 08’ latitude north and between 38^0^ 41’- 40^0^ 44’ longitude east. According to the Central Statistical Agency (CSA), the Bale zone had a total population of 1,840,746, including 932,224 men and 908,522 women in 2017. Of the total inhabitants in Bale Zone, 269,139 (14.62%) were urban dwellers and 1,571,605 (85.38%) were rural residents [40]. The zone comprises 18 districts and a total of 297,081 households (with an average of 4.72 persons per household). There are two administrative towns in the zone namely, Robe and Goba towns. Furthermore, each administrative town is divided into Kebeles (smaller administrative units). Kebeles are further subdivided into smaller clusters known as *‘gots’*. Robe and Goba towns have 36 and 24 *gots* respectively. According to the 2021 administrative report, the population of Robe and Goba towns comprised 73152 and 52785 people respectively.

### Sample size determination and sampling procedures

Based on the total population in the zone, the study sample size of 700 individuals was calculated using OpenEpi (Version 3, an open-source calculator) and considering the following parameters: 95% level of confidence, 4% margin of error, 24.4% reported prevalence of abdominal obesity by the previous study in Dilla town, South Ethiopia [38], design effect of 1.5, and non-response rate of 5%. A multi-stage stratified sampling and, systematic random sampling was employed to select the study participants. Initially, the study population sampling was stratified into the two (Robe and Goba) towns, followed by stratification into randomly selected clusters (*gots*). One-third of clusters were selected from each town, and proportionate to their number, twelve and eight gots were selected from Robe and Goba respectively. Furthermore, households in the sampled clusters were selected using systematic sampling techniques and one adult per sampled household was selected using the lottery method.

### Data collection and measurement procedures

Interviewer-administered, structured questionnaires were used to collect data on socio-demographic and behavioural characteristics followed by physical measurements of weight, height, waist, and hip circumferences. A standard questionnaire was adapted from the WHO STEPS-wise questionnaire for chronic disease risk factor surveillance [41]. The English version of the questionnaire was translated into Afan Oromo and Amharic, the local languages spoken in the study area. After the translation, the questionnaire was -translated back to English to check the consistency. Weight was measured using an electronic digital weighing scale (Healthgenie HD-221) by putting the scale on a firm flat surface. Before taking weight, participants were asked to take off footwear, heavy clothes, and empty their pockets of heavy items. Participants’ height measurements were taken in a standing position by a portable height measuring board placed on a firm surface against the wall. With participants facing the data collector, feet placed together, and eyes leveled at the ears, readings were taken in centimeters (to the nearest 0.1 centimeters). With the participants’ arms relaxed at the sides, the waist circumferences were measured by constant tension tape at the end of a normal expiration; at the midpoint between the lower margin of the last palpable rib and the top of the iliac crest (hip bone). Waist measurements were read at the level of the tape to the nearest 0.1 centimeters while ensuring the tape was comfortably tight enough not to cause compression of the skin. Hip circumferences were measured by constant tension tape with the arms relaxed at the sides at the maximum circumference over the buttocks. Hip circumferences were measured and read at the level of the tape to the nearest 0.1 centimeters. Further details on the study’s physical measurement protocols were consistent with the WHO STEPS-wise instrument guideline [42].

Data collection was conducted by six data collectors (three male-female pairs) with bachelor’s degree in health sciences (Nursing, Public Health, and Midwifery). Female and male data collectors respectively collected female and male participants’ data. Two supervisors with master’s degrees in public health oversaw the data collection process. Data collectors and supervisors were provided with a two-day intensive training on the objective of the study, administration protocols for the questionnaires, administration protocols for anthropometric measurements (weight, height, waist, and hip circumferences), and how to maintain confidentiality and privacy of the study participants. Assisted by the study supervisors, every day before leaving the households (where the data was collected), data collectors checked all the questionnaires for completeness.

### Outcome variable

Derived from waist circumference measurements, central (abdominal) obesity was the dependent variable for the current study. As per the World Health Organization recommendations, waist-circumference >94 centimeters for males and >80 centimeters for females were categorized as central obesity [23]. Individuals with central obesity were coded as “1” and others were coded as “0”.

### Independent variables

Independent variables included sociodemographic and behavioral variables. Sociodemographic variables included the town of residence (Robe or Goba); age (categorized into 18–29, 30–39, 40–49, 50–59, and ≥ 60 years); sex (male or female), marital status (categorized as never married, married/cohabiting, or divorced/separated/widowed); educational status (categorized as no formal education, secondary education, or diploma and above); occupational status (housewives, self-employed, government/non-government/private employee, student/unemployed, or Retired) and family size (categorized as ≤ 2, >2). The wealth index was computed using principal component analysis (PCA), household asset items, and other variables [43, 44]. We examined the assumptions of PCA including: correlation matrix for the variables containing 2 or more correlations ≥ 0.30, variables with measures of sampling adequacy less than 0.50 that must be removed (looking anti-image),the overall measures of sampling adequacy (KMO) ≥ 0.5) [45], Bartlett test of sphericity (p-value < 0.05), having commonality > 0.5, and not having the complex structure (correlation ≥ 0.40). Components that collectively explained more than 60% of the variance in the set of variables and eigenvalues > 1 [46, 47] were used to identify variables to be included in further analyses. Ultimately, the economic status of study subjects was categorized into tertiles as rich, medium, and poor. Behavioral variables included characteristics such as fruit and vegetable consumption. One medium-sized piece of apple, banana, or orange and/or a half cup of chopped or cooked fruit was considered to be one serving. One cup of raw green leafy vegetables and/or half a cup of other vegetables, cooked or chopped raw was considered to be one serving. Categories of fruit and/or vegetables consumption were described as less than five servings of fruit and/or vegetables per day, or five or more servings of fruit and/or vegetables per day. Other food-related variables included the number of days consuming fruits and/or vegetables, skipping breakfast (categorized as yes or no), and avoidance of eating foods prepared outside the home (categorized as yes or no). Levels of physical activities was derived by calculating the metabolic equivalent value (MET) minutes. We used MET-minutes to capture the intensity of physical activity. MET is the ratio of a person’s working metabolic rate relative to the resting metabolic rate. One MET was defined as the energy cost of sitting quietly and is equivalent to a caloric consumption of 1 kcal/kg/hour. It is estimated that, compared to sitting quietly, a person’s caloric consumption is four times as high when being moderately active, and eight times as high when being vigorously active. Therefore, for the calculation of the categorical indicator of the recommended amount of physical activity for health, the total time spent in physical activity during a typical week and the intensity of the physical activity were taken into account. Further details on the calculation of the MET values are consistent with the WHO STEPS-wise instrument guideline [42]. All domains of physical activity including work, transport, and recreation were used for the calculation of MET minutes. Study participants with an equivalent combination of moderate and vigorous-intensity physical activity achieving at least 600 MET-minutes were categorized as having a sufficient level of physical activity while others were categorized as having insufficient physical activity levels. Sedentary activities were measured by adding the total time spent sitting or reclining on a typical day. Current smokers of tobacco were categorized as yes or no and participants who consumed alcohol were categorized as never drank, consumed in the last 12 months, or consumed in the last 30 days. Measurements including fruit and/or vegetable consumption, level of physical activities, alcohol, and/or tobacco consumption were assessed and analyzed using World Health Organization (WHO) STEPS Surveillance tool recommendations. Detailed information has been reported elsewhere [42].

### Data analysis procedures

The data were coded and entered into EpiData Version 3.1 and were cleaned, processed and analyzed using SPSS version 25 and STATA version 14. The study variables were described using mean, frequencies, proportions, and tables. The Chi-square test was used to check the statistical difference in the distribution of categorical independent variables between men and women. A two-sample Wilcoxon rank-sum (Mann-Whitney) test was used to check the statistical difference in the distribution of continuous independent variables between men and women. Both bi-variable and multi-variable binary logistic regression analyses were used to identify factors associated with the outcome variable. Variables having a p-value of less than 0.25 in the bi-variable binary logistic regression model were included in the multivariable binary logistic regression analysis model. The enter method was used to run the model. The logit of the dependent variable was checked for outliers and 7 outlying values (having standardized residual >2.58 at the level of α<0.01) were excluded from the analysis. Hosmer and Lemeshow’s goodness of fit model was checked and the data fitted the model well (p = 0.92). Multi-collinearity between independent variables was checked using the variance inflation factor (VIF), the mean VIF was 2.1 which is less than the recommended cut-off values [48]. Finally, adjusted odds ratios with 95% confidence intervals were used to estimate the strength of associations between the outcome variable and independent variables. All tests were two-tailed and statistical significances were declared at a p-value <0.05.

### Ethical considerations

Ethical clearance and support letters to introduce the researchers and the study to the respective study areas were obtained from the ethical review committee of Madda Walabu University. Permission letters to conduct the survey were obtained from the respective authorities of the two towns (Robe and Goba). The methods were conducted following the tenets of the Helsinki declaration. To obtain oral informed consent an information sheet was read to all eligible study participants before data were collected. The privacy of the respondents was respected and data were de-identified before analysis and were reported in aggregate.

### Inclusivity in global research

Additional information regarding the ethical, cultural, and scientific considerations specific to inclusivity in global research is included in the Supporting Information (S1 Checklist).

## Results

### Sociodemographic characteristics of study participants

A total of 694 adults (259 men and 435 women) participated in this study with a response rate of 99.1%. Four hundred and eight (58.8%) and 286 (41.2%) study participants were from Robe and Goba towns, respectively. Participants’ age ranged from 18–95 years old. Men participants’ median age was 41 years old with an interquartile range (IQR) of 28–55 years. Women’s median age was 32 years with an IQR of 25–45 years (Table 1).

**Table 1:** Sociodemographic characteristics of study participants in administrative towns of Bale Zone, Southeast Ethiopia, 2021.

Variables	Total (%), N = 694	Men (%), n = 259	Women (%), n = 435	Pearson chi square (df)	p-value
**Town of residence**					
Robe	408 (58.8)	161 (62.2)	247 (56.8)	29.2 (4) (1)	<0.001*
Goba	286 (41.2)	98 (37.8)	188 (43.2)		
**Age category**					
18–29 years	262 (37.8)	79 (30.6)	183 (42.2)	29.2 (4)	<0.001*
30–39 years	134 (19.4)	41 (15.9)	93 (21.4)		
40–49 years	103 (14.9)	37 (14.3)	66 (15.2)		
50–59 years	92 (13.3)	52 (20.2)	40 (9.2)		
≥60 years	101(14.6)	49 (19.0)	52 (12.0)		
**Educational status**					
No formal education	98 (14.1)	23 (8.9)	75 (17.2)	33.7 (3)	<0.001*
Primary education (1–8)	205 (29.6)	54 (20.9)	151 (34.7)		
Secondary education (9–12)	219 (31.6)	94 (36.4)	125 (28.74)		
Diploma and above	171 (24.7)	87 (33.7)	84 (19.3)		
**Ethnicity**					
Oromo	546 (78.7)	216 (83.4)	330 (75.9)	4.8 (3)	0.1
Amhara	114 (16.4)	33 (12.8)	81 (18.6)		
Wolaita	11 (1.6)	4 (1.5)	7 (1.6)		
Others^a^	13 (1.9)	4 (1.5)	9 (2.0)		
Refused	10 (1.4)	2 (0.8)	8 (1.8)		
**Religion**					
Muslim	291 (41.9)	116 (44.8)	175 (40.2)	2.79 (3)	0.4
Orthodox Christian	333 (48.0)	119 (46.0)	214 (49.2)		
Protestant Christian	69 (9.9)	23 (8.9)	46 (10.6)		
Catholic	1 (0.1)	1 (0.4)	0 (0.0)		
**Marital status**					
Never married	139 (20.0)	68 (26.3)	71 (16.3)	27.4 (2)	<0.001*
Married/cohabiting	465 (67.0)	177 (68.3)	288 (66.2)		
Divorced/separated/widowed	90 (13.0)	14 (5.4)	76 (17.5)		
**Occupational status**					
Housewives	239 (34.8)	-	239 (55.2)	219.1 (4)	<0.001*
Self-employed	195 (28.4)	112 (44.1)	83 (19.2)		
Government/private/NGO employed	113 (16.5)	69 (27.2)	44 (10.2)		
Retired	38 (5.5)	25 (9.8)	13 (3.0)		
Student/unemployed	102 (14.8)	48 (18.9)	54 (12.5)		
**Family size**					
≤2	391 (56.3)	121(46.7)	270 (62.1)	15.5 (1)	<0.001*
>2	303 (43.7)	138 (53.3)	165 (37.9)		
**Wealth index**					
Low	279 (40.2)	85 (32.8)	194 (44.6)	10.5 (2)	0.005*
Medium	161 (23.2)	63 (24.3)	98 (22.5)		
High	254 (36.6)	111 (42.9)	143 (32.9)		

**Notes**:

^**a**^Somali, Gurage, and Tigre;

*significant at p <0.05

### Behavioral characteristics of the study participants

More than half (58%) of the study participants consumed less than five servings of fruit and/or vegetable per day. The mean number of days when fruit was consumed per week was 2.3 days. Approximately 15% of the study participants reported skipping breakfast and another 15% avoided eating foods prepared outside the home. Nearly 29% of the study participants had an insufficient level of physical activity as per World Health Organization recommendations (Table 2).

**Table 2:** Behavioral characteristics of study participants in administrative towns of Bale zone, Southeast Ethiopia, 2021.

Variables	Total (%), N = 694	Men (%), n = 259	Women (%), n = 435	Pearson chi square (df)	p-value
**Number of days eat fruit and/or vegetables on average per week**					
No fruit and/or vegetable	58 (8.4)	30	28 (6.4)	77 (3)	0.05
1–2	349 (50.4)	118	231(53.1)		
3–4	219 (31.6)	87	132 (30.3)		
> = 5	67 (9.7)	23	44 (10.1)		
**Number of servings of fruit and/or vegetables on average per day**					
1–2 servings	49 (8.1)	17 (8.0)	32 (8.2)	0.4 (2)	0.8
3–4 servings	301 (49.8)	102 (48.3)	199 (50.9)		
> = 5 servings	252 (41.7)	92 (43.6)	160 (40.9)		
**Avoid eating foods prepared outside of a home**					
Yes	584 (84.6)	176 (68.2)	408 (94.4)	85.4 (1)	<0.001*
No	106 (15.4)	82 (31.8)	24 (5.6)		
**Skip breakfast**					
No	592 (14.5)	210 (81.4)	382 (88.0)	5.7 (1)	0.01*
Yes	100 (88.5)	48 (18.6)	52 (12.0)		
**Level of physical activity**					
Insufficient physical activity	195 (28.8)	31 (12.3)	164 (38.6)	53.3 (1)	<0.001*
Sufficient physical activity	482 (71.2)	221 (87.7)	261 (61.4)		
**Number of days fruit consumed in a typical week (mean ± SD)**	2.33 ± 1.60	2.31 ± 1.57	2.35 ± 1.62	Z = 0.1	0.9
**Number of days vegetables consumed in a typical week (mean ± SD)**	3.59 ± 1.97	3.49 ± 1.83	3.66 ± 2.05	Z = -0.45	0.6
**Number of servings of fruit on average per day (mean ± SD)**	2.11 ± 0.86	2.25 ± 0.99	2.04 ± 0.77	Z = 2.28	0.02*
**Number of servings of vegetables on average per day (mean ± SD)**	2.11 ± 0.96	2.11 ± 0.98	2.12 ± 0.95	Z = -0.28	0.7
**Number of servings of fruit and/or vegetables on average per day (mean ± SD)**	4.2 ± 1.40	4.36 ± 1.54	4.14 ±1.31	Z = 1.35	0.1
**Minutes spent on sedentary activities on average per day (mean ± SD)**	178.11 ± 128.98	166.65 ± 125.25	184.90 ± 130.80	Z = -1.78	0.07

**Notes**: Z, critical value for two-sample Wilcoxon rank-sum (Mann-Whitney) test;

*significant at p <0.05

### Alcohol and tobacco use behavioural characteristics of the study participants

Only 7 (1%) participants indicated currently smoking tobacco products and 222 (32.0%) consumed alcohol in the past twelve months (Table 3).

**Table 3:** Alcohol and tobacco consumption behavior of study participants in administrative towns of Bale zone, Southeast Ethiopia, 2021.

Variables	Total (%), N = 694	Men (%), n = 259	Women (%), n = 435	Pearson chi square (df)	p-value
**Currently smoke tobacco products, n = 694**					
Yes	7 (1.0)	7 (2.7)	0 (0.0)	11.87 (1)	<0.001*
No	687 (99.0)	252 (97.3)	435 (100)		
**Ever consumed alcohol, n = 693**					
Yes	222 (32.0)	94 (36.4)	128 (29.4)	3.65 (1)	0.05
No	471 (68.0)	164 (63.6)	307 (70.6)		
**Consumed alcohol in the past 12 months, n = 222**					
Yes	212 (95.5)	90 (94.7)	122 (96.1)	0.22 (1)	0.6
No	10 (4.5)	5 (5.3)	5 (3.9)		
**Frequency of alcohol drinking in the past 12 months, n = 222**					
5–6 days per week	1 (0.5)	0 (0.0)	1 (0.8)	38.50 (3)	<0.001*
1–4 days per week	25 (11.7)	18 (20.0)	7 (5.7)		
1–3 days per week	89 (41.8)	52 (57.8)	37 (30.1)		
Less than once a month	97 (46.0)	20 (22.2)	78 (63.4)		
**Consumed alcohol within the past 30 days, n = 222**					
Yes	134 (60.5)	68 (71.6)	67 (52.3)	8.44 (1)	0.004*
no	88 (39.5)	27 (28.4)	61 (47.7)		
**Five or more drinks on a single occasion at least once during the past 30 days, n = 135**					
Yes	23 (17.0)	15 (5.8)	8 (1.8)	7.91 (1)	0.005*
No	112 (83.0)	244 (94.2)	427 (98.2)		
**Number of drinking occasions in the past 30 days among current (30 days) drinkers (mean ± SD), n = 134**	2.53 ± 1.96	3.13 ± 2.14	1.92 ± 1.54	Z = 4.63	<0.001*
**Number of standard drinks per drinking occasion among current (past 30 days) drinkers (mean ± SD), n = 133**	2.35 ± 1.20	2.73 ± 1.29	1.97 ± 0.98	Z = 3.77	<0.001*
**Maximum number of standard drinks consumed on one occasion in the past 30 days (mean ± SD), n = 134)**	2.87 ± .47	3.52 ± 1.63	2.24 ± 0.96	Z = 5.33	<0.001*

**Notes**: Z, critical value for two-sample Wilcoxon rank-sum (Mann-Whitney) test;

*significant at p <0.05

### Prevalence of central obesity

As described in Table 4, the overall prevalence of central obesity was 39.01% (95% CI: 35.36–42.76). Of the 259 men included in this study, 40 (15.44%, 95% CI: 11.26–20.43%) had waist circumference >94 centimeters. Of the 433 women included in the study, 230 (53.12%, 95% CI: 48.29–57.89%) had a waist circumference greater than 80 centimeters. Based on the waist-to-hip ratios, 204 (47.11%, 95% CI: 42.32, 51.93%) and 129 (49.80%, 95% CI: 43.55–56.06%) women and men had wait to hip ratios of greater than or equal to 0.85 and 0.90, respectively.

**Table 4:** Distribution of central obesity by sex among adult populations in administrative towns of Bale zone, Southeast Ethiopia, 2021.

Central obesity distribution by sex	Frequency	Percent (95% CI)
**Men (n = 259)**		
Waist circumference > 94 centimeters	40	15.44 (11.26–20.43)
Waist circumference > 102 centimeters	9	3.47 (1.60–6.49)
Waist to hip ratio ≥ 0.9	129	49.81 (43.55–56.06)
**Women (n = 433)**		
Waist circumference > 80 centimeters	230	53.12 (48.29–57.89)
Waist circumference > 88 centimeters	118	27.19 (23.11–31.70)
Waist to hip ratio ≥ 0.85	204	47.11 (42.32–51.93)
^a^ **Overall (both sexes) N = 692**		
Centrally obese	270	39.01 (35.36–42.76)

Notes:

^**a**^ calculated using waist circumference >94 and >80 centimeters for men and women respectively.

### Factors associated with central (abdominal) obesity

Findings of the multivariable binary logistic regression analysis revealed that women had higher odds of central obesity compared to men (AOR: 12.93, 95% CI: 6.74–24.79). Age groups 30–39 (AOR: 2.80, 95% CI: 1.59–4.94), 40–49 (AOR: 7.66, 95% CI: 3.87–15.15), 50–59 (AOR: 4.65, 95% CI: 2.19–9.89), and ≥60 years (AOR: 12.67 95% CI: 5.46–29.39) had higher odds of central obesity compared to those below 30 years old. Likewise, Government/Non-governmental/Private employees (AOR: 4.68, 95% CI: 1.47–14.88), self-employed (AOR: 4.63, 95% CI: 1.62–13.24), and housewives (AOR: 5.21, 95% CI: 1.85–14.62) were more likely to be centrally obese compared to unemployed or students. Moreover, individuals who skipped breakfast were at a 54% reduced risk of central obesity compared to their counterparts (Table 5).

**Table 5:** Factors associated with central obesity among adult men in administrative towns of Bale zone, Southeast Ethiopia, 2021.

Variables	Abdominal obesity		COR (95% CI)	AOR^a^ (95% CI)
	Yes	No		
	n (%)	n (%)		
**Residential town**				
Goba	124 (43.4)	162 (56.6)	1.4 (1.0–1.8)	1.10 (0.72–1.70)
Robe	146 (36.0)	260 (64.0)	**1**	**1**
**Sex**				
Male	40 (15.4)	219 (84.6)	**1**	**1**
Female	230 (53.1)	203 (46.9)	6.2 (4.2–9.1)*	12.93 (6.74–24.79)**
**Age in years**				
18–29	56 (21.4)	206 (78.6)	**1**	**1**
30–39	59 (44.4)	74 (55.6)	2.9 (1.9–4.6)*	2.80 (1.59–4.94)**
40–49	61 (59.2)	42 (40.8)	5.3 (3.3–8.7)*	7.66 (3.87–15.15)**
50–59	37 (40.2)	55 (59.8)	2.5 (1.5–4.1)*	4.65 (2.19–9.89)**
> = 60	56 (56.0)	44 (44.0)	4.7 (2.9–7.7)*	12.67 (5.46–29.39)**
**Marital status**				
Never married	19 (13.7)	120 (86.3)	**1**	1
Married/cohabiting	195 (42.1)	268 (57.9)	4.6 (2.7–7.7)*	1.38 (0.63–3.05)
Divorced/separated/widowed	56 (62.2)	34 (37.8)	10.4 (5.5–19.8)*	1.05 (0.40–2.78)
**Educational status**				
No formal education	62 (63.3)	36 (36.7)	4.9 (2.9–8.5)*	1.66 (0.70–3.90)
Primary education (1–8)	100 (49.3)	103 (50.7)	2.8 (1.8–4.3)*	1.38 (0.68–2.80)
Secondary education (9–12)	64 (29.2)	155 (70.8)	1.2 (0.8–1.9)	0.94 (0.48–1.86)
Diploma and above	44 (25.7)	127 (74.3)	**1**	**1**
**Occupational status**				
House wives	154 (61.1)	98 (38.9)	11.7 (6.0–22.4)*	5.21 (1.85–14.62)**
Self employed	57 (31.0)	127 (69.0)	3.3 (1.7–6.6)*	4.63 (1.62–13.24)**
Government/private/NGO employed	33 (29.2)	80 (70.8)	3.1 (1.5–6.3)*	4.68 (1.47–14.88)**
Retired	12 (33.3)	24 (66.7)	3.7 (1.5–9.3)*	2.78 (0.73–10.48)
Student/unemployed	12 (11.9)	89 (88.1)	**1**	**1**
**Family size**				
≤ 2	28 (40.0)	42 (60.0)	**1**	-
> 2	184 (37.9)	301 (62.1)	0.9 (0.5–1.5)	-
**Wealth index**				
Low	105 (41.3)	173(58.7)	**1**	**1**
Medium	60 (37.5)	100 (62.5)	0.9 (0.8–1.6)	0.69 (0.40–1.17)
High	105 (41.3)	149 (58.7)	1.2 (0.8–1.6)	0.74 (0.45–1.22)
**Fruit and/or vegetable consumption on average per day**				
Less than five servings per day	128 (36.4)	224 (63.6)	0.8 (0.5–1.1)	0.87 (0.57–1.32)
Five or more servings per day	108 (43.0)	143 (57.0)	**1**	**1**
**Avoid eating foods prepared outside of a home**				
Yes	245 (42.1)	337 (57.9)	1	**1**
No	21 (19.8)	85 (80.2)	0.3 (0.2–0.6)*	1.59 (0.79–3.18)
**Skip breakfast**				
No	242 (41.0)	348 (59.0)	**1**	**1**
Yes	27 (27.0)	73 (73.0)	0.1 (0.3–0.9)*	0.46 (0.23–0.90)**
**Level of physical activity**				
Insufficient physical activity	96 (49.5)	98 (50.5)	1.9 (1.3–2.6)*	0.84 (0.55–1.30)
Sufficient physical activity	166 (34.5)	315 (65.5)	**1**	**1**

**Notes**: **n**, frequency; **COR**, crude odds ratio; **AOR**, adjusted odds ratio; **CI**, confidence interval;

^**a**^The model was adjusted for: residence, sex, age, marital status, education status, occupation, fruit and vegetable consumption, avoiding eating foods prepared outside the home, skipping breakfast and level of physical activity;

*significant at p < 0.05 (crude);

**significant at p < 0.05 (adjusted).

## Discussion

The findings of the current study reveal that the overall prevalence of central (abdominal) obesity was 39.0% (95% CI: 35.36–42.76). In comparison to men, women had a higher prevalence of central obesity (53.1% vs 15.4%). The figures reported in this study are comparable with studies conducted in Gondar and Dabat towns, Northwest Ethiopia (37.6% & 33.6%) [37, 49], but higher than the studies conducted in Nekemte town, West Ethiopia (28.4%) [35], Woldia town, Northeast Ethiopia (15.5%) [36], and Dilla town South Ethiopia (24.4%) [38]. The possible variations in the obesity prevalence could be explained by the use of different cutoff values for waist circumferences [36], and the age distribution variation among the study participants [35, 38]. Furthermore, variation in the gender make-up of the samples might be a possible reason for the differences, given such large differences in prevalence found in this study. Though there are slight variations in the prevalence rates across the regions, the magnitude of central obesity in the study appeared to be high. A recent systematic review and meta-analysis in Ethiopia reported that the prevalence of overweight and obesity is increasing especially in urban settings [50]. The results of the present study confirm these findings and suggest gender as a particularly important factor to examine when estimating overall prevalence rates in Ethiopian regions.

In this study, women had 13 times higher odds of central obesity compared to men. This finding is corroborated by similar studies in different parts of Ethiopia [35–39]. Similarly, studies conducted by *Jaacks et al*., suggested that in countries with stage 1 obesity transition, the prevalence of obesity is higher among women compared to men [51]. According to a study conducted by the Global Burden of Diseases researchers, the trend of obesity and overweight is persistently higher among women than men in developing countries [4]. Further appropriately powered longitudinal studies within males and females in Ethiopia examining predictors of obesity/central obesity are warranted.

The current study revealed that the odds of central obesity tend to increase as age increases. Elsewhere, a longitudinal study conducted by *Baum & Ruhm* reported that body weight increased as age increased [52]. The positive association between age and abdominal obesity is supported by several other studies conducted elsewhere including in Ethiopia, China, Russian Federation [35–37, 39, 53–56]. This association might be explained partly by the gradual decline in physiologic activities and basal metabolic rate as age increases [57].

Employed adults and housewives had higher odds of central obesity compared to unemployed or students. This might be explained by the hypothesis that employed adults might have better access to foods than their counterparts. Previous studies in Ethiopia suggested that unemployed men are less likely to be obese compared to employed men [58]. This finding contradicts a study conducted in North Glasgow, UK, which reported that unemployed men and women were less likely to be centrally obese compared to full-time workers [59]. Additional longitudinal epidemiological studies exploring the role of employment status on the development of abdominal obesity in low-income countries are warranted. With regards to findings of the higher odds of central obesity among housewives, gender may play a role here as well. Furthermore, it is possible that that women who stay at home have food within easy reach. Moreover, frequency of meal intakes has been associated with an increase in total energy intake [60], hence increased opportunities to develop obesity.

Individuals who reported skipping their breakfast were 54% less likely to be centrally obese compared to their counterparts. This might be partly explained by the fact that the frequency of meals is associated with an increase in total energy intake [60]. However, this finding is in contrast with a systematic review and meta-analysis conducted by *Ma* et al., which reported that skipping breakfast resulted in a 31% increase in abdominal obesity [61]. In our study, breakfast skipping behavior was measured through self-reporting and the reason for skipping breakfast was not collected. Furthermore, meal frequency and portion size were not assessed, which could be a source of variation with existing evidence. Furthermore, this study was not topic-specific to assess the association between skipping breakfast and the risk of central obesity.

This study aimed to examine predictors of central (abdominal) obesity in a region with no prior evidence on the distribution and associated risk factors. Although the study used primary data on physical measurements like waist and hip circumferences and the WHO-STEPS wise tool for non-communicable disease risk factors surveillance with trained data collectors and supervisors, the findings must be interpreted in light of the following limitations. Firstly, due to the cross-sectional nature of the study, a cause-effect relationship cannot be established between the risk factors and obesity. Secondly, the study sample was too small for sex and age-specific reporting of the prevalence and risk factors. Differences in prevalence and risk factors by gender suggest the need for gender-specific stratification in larger, appropriately powered longitudinal studies. Lastly, data regarding dietary habits, physical activity, alcohol, and tobacco use were collected through self-reported behavior questionnaires, which might be affected by the recall and social desirability bias. Future studies could use more objective measurements, including third part reporting and wearables to assess these behaviors.

## Conclusions

The burden of abdominal obesity is high in the administrative towns of Bale Zone, southeast Ethiopia. One out of every two women and one out of every five men were found to be centrally obese. Female sex, age, and being employed were significantly associated with central obesity, while skipping breakfast was a protective factor. Prevention strategies might be more effective when they occur as part of workplace health promotion programs. Public health campaigns and community health promotion programs directed at women and older adults may also be a valuable use of available resources in order to curb the increasing burden of central obesity and its impact. Further studies of gender-specific risk factors are also warranted to determine the specific associated risk factors and assist in improved tailoring of prevention programs in LMICs.

## Supporting information

S1 Checklist Inclusivity in global research checklist. (DOCX)

S1 File (SAV)
